# A device that facilitates screwing at an appropriate angle in quadrilateral surface fractures: 105-degree drill attachment

**DOI:** 10.55730/1300-0144.5378

**Published:** 2022-02-27

**Authors:** Emre GÜLTAÇ, Cem Yalın KILINÇ, Fatih İlker CAN, İsmail Gökhan ŞAHİN, Ahmet Emrah AÇAN, Çağatay GEMCİ, Nevres Hürriyet AYDOĞAN

**Affiliations:** 1 Department of Orthopedics and Traumatology, Faculty of Medicine, Muğla Sıtkı Koçman University, Muğla, Turkey; 2 Department of Orthopedics and Traumatology, Faculty of Medicine, Balıkesir University, Balıkesir, Turkey

**Keywords:** 105° drill attachment, deep pelvic fractures, acetabular fractures, quadrilateral plate fractures, functional and radiological outcomes

## Abstract

**Background/aim:**

Within this study, we aimed to investigate the radiological and functional outcomes of acetabular fractures involving quadrilateral surface using 105° drill attachment in the anterior intrapelvic approach.

**Materials and methods:**

The 35 patients who underwent surgical treatment from January 2016 and January 2020 for acetabular fractures involving quadrilateral surface with anterior intrapelvic approach using 105° drill attachment and a minimum of 12 months of postoperative follow-up were included. Perioperative complications, operation duration, and the quality of reduction were evaluated. Reduction quality was classified as poor, imperfect, and anatomic. Functional evaluation was performed according to the Harris Hip Score (HHS) and Merle d’Aubigne Score.

**Results:**

Among 35 patients (median age 36 (21–80)), radiological results of the acetabular fixations were anatomic, imperfect, and poor in 28 (80%), 5 (14.3%), and 2 (5.7%) patients, respectively. Postoperative 1-year functional outcomes with Merle d’Aubigne scores and HHS were median 18 (10–18) and 90 (60–96), respectively. The clinical outcomes of the patients showed concordance with reduction quality. The median operation duration was 180 minutes (range 125–270). Iatrogenic neurovascular damage was not noted in any patients.

**Conclusion:**

Reduction and fixation of deep intrapelvic fractures are risky and difficult due to the narrow anatomy and adjacent crucial neurovascular structures. As the 105-degree drill application is safe and easy to intervene in, short surgery duration and satisfactory results with minimum complications can be obtained with a 105 angulated drill in the deep pelvic region.

## 1. Introduction

Anterior, posterior, extensive, or combined approaches are current options for the surgery of acetabular fractures. The modified Stoppa approach has been used more widely in recent years [1]. This approach can be used for fixation of the anterior wall and column, quadrilateral region, and both columns [2]. This approach has extended the possible fixation alternatives by enabling the use of long and vertical infrapectineal plates in addition to classically located suprapectineal reconstruction plates [3]. Fixation of quadrilateral and ischial fractures is possible using the Stoppa approach [4]; however, reduction and fixation might be challenging in such a deep location and also less bone thickness and fragmented fractures may complicate the procedure [5,6]. Today, various implants are available for displaced quadrilateral surface fractures. These implants require further surgical experience [7].

Reduction and fixation of fractures of intrapelvic deep localized areas such as the quadrilateral region and ischial region are riskier and more difficult due to surrounding anatomical structures [6, 8]. Care should be taken to protect all neurovascular structures, primarily including corona mortis; obturator artery, vein, and nerve; iliac artery and vein; and deep pelvic veins which have high risks of bleeding and morbidity [1]. In fractures that require infrapectineal plating or in the use of implants used for quadrilateral surface, it is occasionally difficult to fix screws at the appropriate angle with conventional drills and there is a high risk of injury to surrounding anatomical structures. In addition, even with flexible drills, it is usually challenging to adjust the angle of the drill bit and also, during the rotation of the power driller, the flexible body can wrap the surrounding soft tissue such as surrounding neurovascular structures and bladder. With the usage of the 105-degree drill attachment (Milwaukee, Brookfield, USA, 105° right angle driver extension power screwdriver drill bit attachment) surrounding soft tissue protection and drilling at the appropriate angle can be perfectly achieved. The outer part of the body is immobile during drilling and the inner part is rotating, hence the surrounding soft tissue is protected. The distal part of the drill is the mobile component and the only part that needs attention during the bone drilling. 105-degree angled structure of the drill allows directing the drill bit at the most appropriate angle, especially for screw insertion into the quadrilateral surface in the deep intrapelvic area.

This study aims to assess the effectiveness and outcomes of a new surgical drilling technique with the usage of the 105-degrees drill attachment in deep pelvic fractures by investigating the postoperative functional and radiological results of cases who had at least 1-year follow-up data.

## 2. Materials and Methods

**Figure 1:**
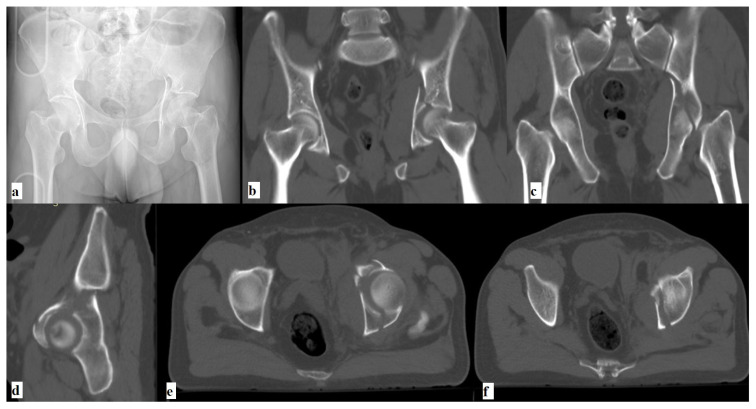
**(a)** Preoperative radiographs and **(b,c,d,e,f)** CT images of a 51 year old male patient who suffered from a traffic accident showing left anterior column/posterior hemitransverse acetabular fracture with acetabulum posterior wall fracture.

**Figure 2:**
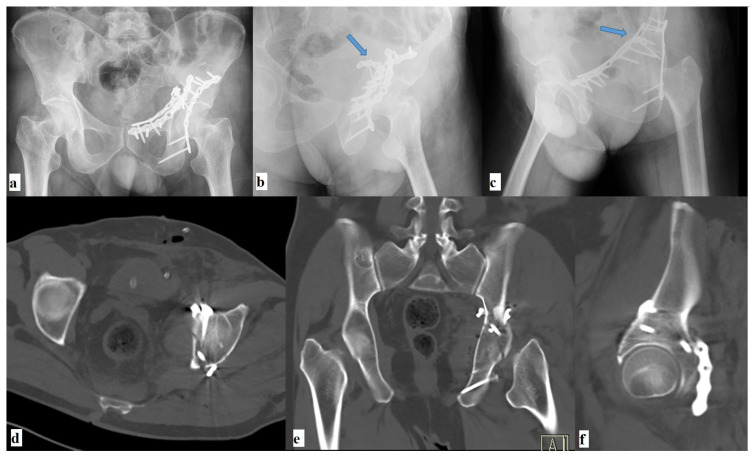
**(a,b,c)** Postoperative radiographs and **(d,e,f)** CT images of the same patient. The blue arrows show the screws placed using the 105-degree drill attachment.

A retrospective study was performed with the approval of institutional review board approval. A retrospective analysis was carried out of 230 patients with acetabular fractures between January 2016 and January 2020. All data were collected from the electronic data archives, operational notes, and radiographs. Variables such as fixation method, surgery duration, and postoperative complications were recorded. One hundred and fifty-three of 230 patients who were operated with anterior intrapelvic approach were investigated in terms of usage of 105-degrees drill attachment and quadrilateral surface involvement. The inclusion criteria were; patients suffering from both column acetabular fractures involving quadrilateral surface and operated with anterior intrapelvic approach, who have complete physical and radiological examination data records of minimum 12 months of postoperative follow-up. Patients with insufficient data in the medical records, concomitant injuries such as femur or tibia fractures in the ipsilateral lower extremity, pediatric cases, history of hip surgery, open injuries in the pelvic region, and patients who were operated on without usage of 105-degrees drill were excluded from the study. Patients with severe comorbid diseases or conditions which made postoperative compliance unreliable were also excluded. After excluded cases, 35 patients who were operated for acetabular fracture involving quadrilateral surface with anterior intrapelvic approach with the usage of 105-degrees drill bit attachment and who had at least 12 months of postoperative follow-up data were included in the study (Figure 1a,1b,1c,1d,1e,1f,2a,2b,2c,2d,2e,2f).

In our clinic, all pelvic fracture surgeries are performed by two experienced surgeons (C.Y.K., E.G.). In the anterior intrapelvic approach, a vertical midline incision is preferred. After the anterior rectus fascia and rectus abdominis are deepened from the midline, the bladder is protected and the fracture line is reached by using blunt finger dissection on the corresponding pelvic side. Thus, a clear field of view of the quadrilateral surface is obtained. During the fixation of fractures in the deep intrapelvic region such as quadrilateral surface fractures we use 105-degree drill attachment instead of conventional drills in order to provide the safest and most appropriate screw angle.

The patients were followed-up for at least 12 months postoperatively. The median follow-up time was 20 months (12–35). On postoperative day 1; anteroposterior and Judet radiographs, pelvic CT scans were investigated to assess the reduction quality of the fractures by a radiologist experienced in extremities, and the fracture reduction was graded as anatomical (≤1mm displacement), imperfect (>1 to <3 mm displacement), or poor (≥3mm displacement) according to the criteria described by Matta [9,10]. The clinical follow-up was planned for 2 weeks, 1 month, 3 months, 6 months, and 1 year postoperatively. Functional results were investigated according to the Harris Hip Score (HHS) [11] and Merle d’Aubigne and Postel Scoring System [12]. Radiographic and functional outcomes were classified as excellent = 4, good = 3, fair = 2, or poor = 1.

### 2.1. Statistical analysis

The statistical analysis was performed by using SPSS version 22.0 statistical software (SPSS Inc., Chicago, Illinois, USA). The data were analyzed with the Shapiro-Wilk test in terms of distribution pattern. Kruskal-Wallis and Pairwise comparison tests were used for comparison of the nonnormally distributed continuous data. The median, minimum, and maximum values of the data were determined with descriptive analysis.

## 3. Results

Among the 35 patients, the median age was 36 (21–80). Median follow-up time was 20 months (12–35). Most of the patients were exposed to high energy trauma, such as motor vehicle accident (60%) and fall from height (%25.7).

Patients were closely monitored and the timing of the operation was decided due to patients’ clinical stability. In the preoperative period, patients’ comorbid diseases and other concomitant trauma-related problems were consulted to the necessary departments. The median time from trauma to surgery was 5 days (0–9). The median operation duration was 180 min (125–270).

The reduction quality of acetabular fractures on postoperative day 1 was graded as anatomic, imperfect, and poor in 28 (80%), 5 (14.3%), and 2 (5.7%), respectively. According to the Harris Hip Score System (HHS); patients had a median of 90 (60–96) (excellent in 20 patients, good in 10 patients, fair in 3 patients, and poor in 2 patients) (Table 1). Due to Merle d’Aubigne and Postel Scoring System, the median was 18 (10–18) (excellent in 18 patients, good in 12 patients, fair in 3 patients, and poor in 2 patients) (Table 2). In terms of the Merle d’Aubigne Score, there was a significant difference between stage 1 and stage 3 (p = 0.014), while there was a significant difference between stage 1 and stage 2 (p = 0.006). There was no significant difference between stages 2 and 3 in terms of this scoring system. In terms of Harris hips score, there was a significant difference between the patients whose reduction quality was stage 1 and those with stage 3 (p = 0.024), while there was a significant difference between stage 1 and stage 2 (p = 0.010). No statistically significant difference was observed between stages 2 and 3 in this group. Also, there was no statistically significant difference between the groups in terms of the operation time, follow-up time, and time to surgery. Two patients had poor clinical outcomes and these patients were over the age of 65 years and had poor and imperfect reduction quality, respectively. These patients underwent total hip arthroplasty afterward.

Iatrogenic neurovascular damage and postoperative deep infection were not noted in any patients operated with a 105-degrees drill attachment. There was no articular screw penetration in postoperative CT scans. Heterotrophic ossification was also not observed in any of the patients. Postoperative deep vein thrombosis developed in 2 patients with comorbid diseases.

## 4. Discussion

Because of the intra-articular nature of the acetabular fractures, the main objectives of surgical treatment are to provide anatomical reduction without articular step-off and achieve stable fixation, to start joint movements in early stages, and to gain joint functions as soon as possible. Studies have shown that even millimetric displacement may result in progressive posttraumatic osteoarthritis and that clinical and radiological results will not be satisfactory [9,10]. However, ensuring anatomical reduction is often difficult due to the three-dimensional complex anatomy of the acetabulum and pelvis [13]. A wide variety of reduction and fixation materials can be used during surgery. However, it may not be possible to orient the screw direction at the desired angle in the deep pelvic region. The main difficulties are often accompanied by medial protrusion of the femoral head, a high degree of disintegration, as well as dome impaction [14]. Pelvic brim plates are successful in preventing medial displacement, but it is quite difficult to insert the screws into these plates periarticularly [15]. The application of pelvic brim plate was first mentioned by Hirvensalo et al [16]. Later, an infrapectineal plate was described by Cole and Bolhofner for the treatment of acetabular fractures involving quadrilateral surface with a modified Stoppa approach [1]. They stated that one should be careful not to place the screws directly adjacent to the quadrilateral surface as the screws may penetrate the joint. The authors also indicated that care should be taken to protect all neurovascular structures, primarily including corona mortis; obturator artery, vein, and nerve; iliac artery and vein; and deep pelvic veins which have high risks of abundant bleeding and morbidity. In addition, acetabular fracture surgery requires surgical specialty due to the complex pelvic anatomy and therefore it has a prolonged and steep learning curve [17–19].

**Figure 3:**
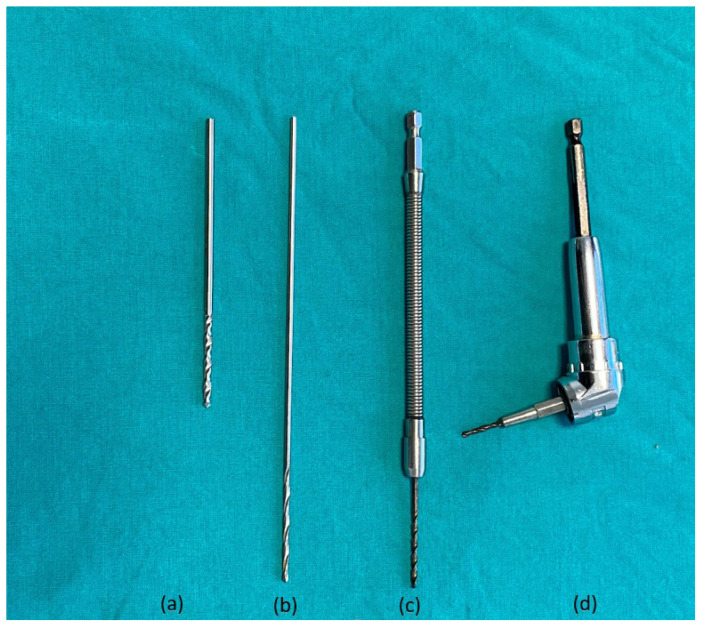
**(a)**Short conventional drill attachment, **(b)** Long conventional drill bit, **(c)** Flexible drill bit, **(d)** 105-degree drill bit attachment.

**Figure 4:**
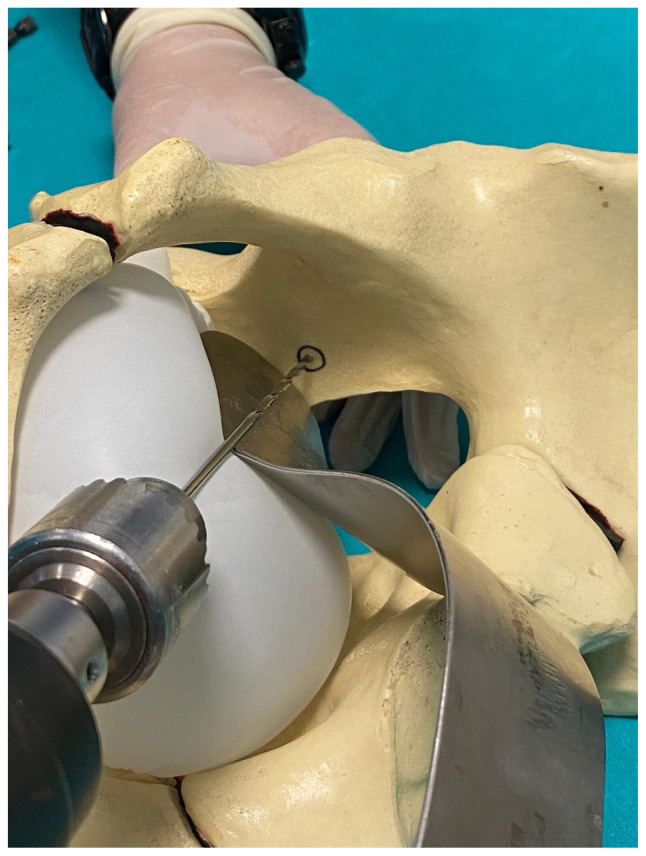
Conventional straight drill. Due to the shape of the conventional drill, the screw cannot always be sent at the appropriate angle. In the deep intrapelvic region (figure 4), it is only possible to insert a screw oriented inferiorly.

**Figure 5:**
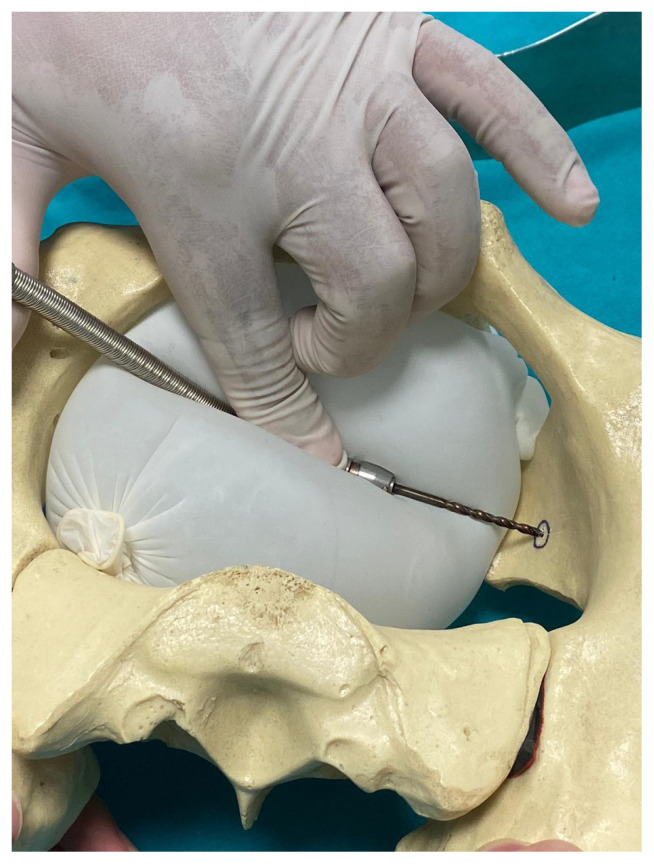
Conventional flexible drill. The surgeon using the flexible drill has to hold the drill in the desired axis with one hand as shown in Figure 5 and it may cause damage to the surrounding soft tissues such as the bladder.

**Figure 6:**
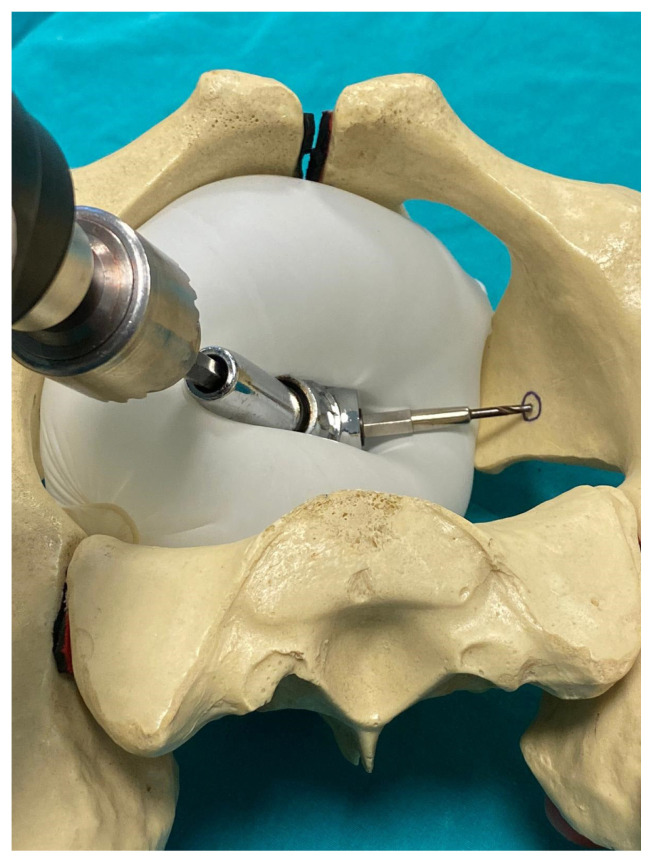
105-degree drill attachment. This drill allows the surgeon to screw in the appropriate axis without having to hold it with another hand and prevents damage to the surrounding soft tissues.

**Figure 7:**
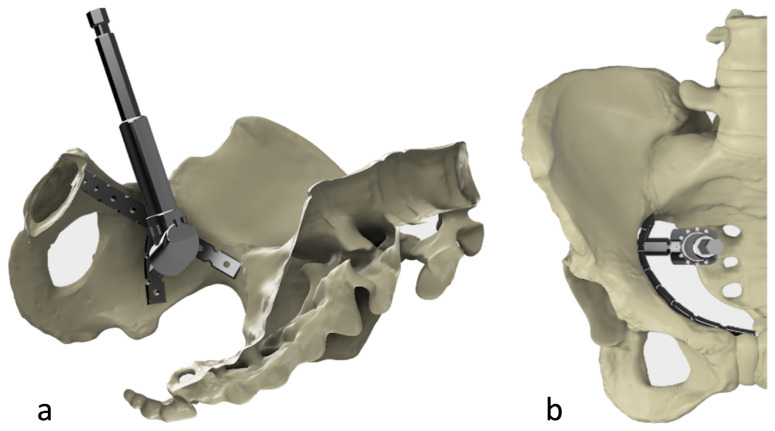
**(a)** Usage of 105-degree drill attachment at quadrilateral surface of the inner pelvis **(b)** The schema of the superior view during usage of 105-degree drill attachment at quadrilateral surface of inner pelvis.

Fixation of fractures of the quadrilateral surface is challenging due to the position of the plate in the lesser pelvis [6, 8]. It is difficult to drill holes in the appropriate direction from the deep pelvic region when drilling holes in plates located in the deep pelvic region. There are various drills that can be used in these surgeries (Figure 3a,3b,3c,3d). Especially when using a conventional drill, adjusting the drill orientation is often difficult due to both bladder and retractors (Figure 4). Flexible drilling requires both hands in order to position the drill bit at the appropriate angle, hence it is difficult to use both hands in a narrow deep pelvic region (Figure 5). With our new drilling technique, we use the advantage of being able to fixate using one hand. And also when using a flexible drill, the body of the drill can wrap the surrounding soft tissue. The use of the 105° angle drill allows the screws to be placed at any appropriate angle in the deep intrapelvic region. Another important issue is that the body part of the drill is protected and minimizes the possibility of winding in the surrounding tissues during the drilling process (Figure 6, 7a,7b). Due to the accumulation of abdominal subcutaneous fat tissue, management of acetabulum fractures in a deeper hole are more challenging in obese patients. Additional assistant surgeons and special surgical equipment are often needed to aid in soft tissue retraction [6]. The 105° angle drill gains more importance in obese patients.

T-shaped, anterior column and posterior hemitransverse, both columns, posterior column, and combined transverse fractures are most often associated with medial migration of the quadrilateral regions [20,21]. In our study, in order to standardize fractures, we only investigated patients with both column fractures involving quadrilateral surface.

In the literature, there are many studies reporting satisfying outcomes. Sagi et al. reported that excellent/good acetabular fracture reduction was achieved in 92% of 57 cases and clinical outcomes were excellent/good (91 %) according to Merle d’Aubigne Score [22]. Isaacson et al. reported an anatomic or good reduction in 92% of 36 cases and good/excellent clinical results, with a Merle d’Aubigne score of 82% [23]. Hirvensalo et al. reported excellent or satisfactory fracture reduction quality in 84% of a series of 164 cases and good/excellent HHS results in 75% of cases [16]. Liu et al. reported 16 excellent or good fracture reduction that was obtained in 92% of 24 cases, and good/excellent clinical HHS results in 93% [3].

Correspondingly, in our study, the anatomic reduction was achieved in 28 (80%) patients. The clinical results were excellent/good in 30 (85.7%) cases according to Modified Merle d’Aubigne Score System and similarly, according to HHS, excellent/good results were investigated in 30 (85.7%) cases. The clinical outcomes of the patients showed concordance with reduction quality according to our clinical experience.

**Figure 8:**
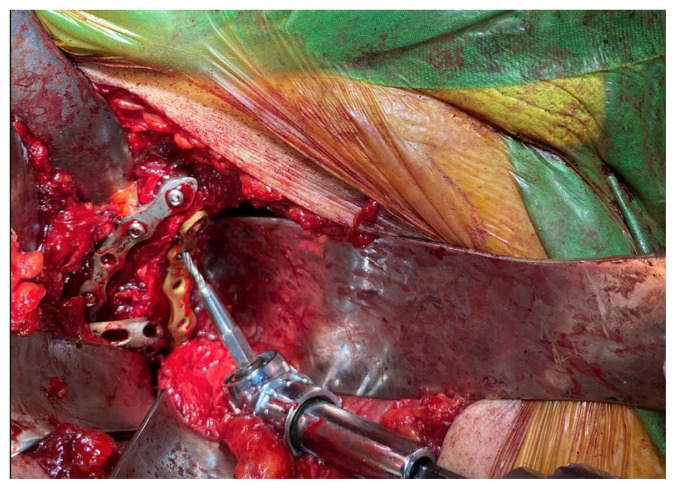
Intraoperative photograph showing usage of 105-degree drill attachment at quadrilateral surface of inner pelvis.

In our clinic, we also use conventional and flexible drills but especially in fractures of the deep intrapelvic region we use a 105-degree drill that can be used with different size drills in the deep intrapelvic region (Figure 8). In this study, there was no articular penetration of a screw in postoperative control CT scans. Neurovascular damage was not noted in any cases operated with a 105-degrees drill attachment. When the literature is investigated, contralateral results have been reported in terms of neurological damage. Sagi et al. reported paralyzed obturator nerve in 13 patients (26%) postoperatively [22]. Also, Ma et al. and Laflamme et al. reported 2 (6.7%) and 1 (4.8%) patients with obturator nerve palsy after acetabular surgery with Modified Stoppa approach, respectively [5,24]. Except for preserving the joint from screw penetration, we observed that the biggest advantage of the 105-degree drill is being more effective in protecting the neurovascular structures.

Sagi et al. conducted a study with a group of 57 patients operated with an anterior intrapelvic approach and reported a mean operation time of 263 min [22]. In our study, we observed that the median operation time was 180 min (125–270). We think that the operation duration is shorter when compared to the literature findings as it enables fixation at one time and in the most appropriate screw position without multiple drills.

Sagi et al. reported deep infection in 1 patient (1.8%), while Cole et al. reported 1 patient (1.8%) and Hirvensalo et al. reported 5 patients who had a deep infection (3%) [1,16,22]. In our study, deep infection was not observed in any patients. We think that the short operation time achieved with the 105-degree drill attachment technique and compliance with surgical sterilization rules in acetabular surgery operations in our clinic enabled us to achieve this result.

The small sample size, the retrospective nature of our study, the absence of a control group, and the inability to compare the results and surgical duration of similar patients treated with conventional drill were the major limitations. The absence of patient-related health quality of life measurement is also a limitation of the study.

## 5. Conclusion

According to our clinical observations, with the usage of 105-degree drill attachment, radiological and clinical results were gratifying whereas complication rates were significantly less. Using a 105-degree drill attachment in deep intrapelvic fractures, which are difficult to intervene like the quadrilateral surface, allows drilling and screw insertion at the most appropriate angle, shortening the operation time and protecting the surrounding soft tissues.

## Figures and Tables

**Table 1:** Radiological results (quality of reduction) according to Matta’s Radiologic Criteria and clinical results according to Harris Hip Score.

		Matta’s Reduction Criteria			
Harris Hip Score		Anatomical	Imperfect	Poor	
	Excellent	18	2	0	20 (57.1%)
	Good	8	2	0	10 (28.6%)
	Fair	2	1	0	3 (8.6%)
	Poor	0	0	2	2 (5.7%)
		28 (80%)	5 (14.3%)	2 (5.7%)	

**Table 2:** Radiological results (quality of reduction) according to Matta’s Radiologic Criteria and clinical results according to Merle d’Aubigne-Postel Scoring System.

		Matta’s Reduction Criteria			
m Merle d’Aubigne-Postel Score System		Anatomical	Imperfect	Poor	
	Excellent	16	2	0	18 (51.4%)
	Good	10	2	0	12 (34.3%)
	Fair	2	1	0	3 (8.6%)
	Poor	0	0	2	2 (5.7%)
		2(80%)	5 (14.3%)	2 (5.7%)	
